# Assessment of changes in the prevalence of personality disorders admitted for psychiatric hospitalization in years 2009-2021

**DOI:** 10.1192/j.eurpsy.2023.419

**Published:** 2023-07-19

**Authors:** A. Gabryelska, M. Sochal, D. Strzelecki

**Affiliations:** ^1^Department of Sleep Medicine and Metabolic Disorders; ^2^Department of Affective and Psychotic Disorders, Medical Univesity of Lodz, Lodz, Poland

## Abstract

**Introduction:**

Personality disorder (PD) is defined as an enduring and inflexible pattern of long duration leading to significant distress or impairment and is not due to the use of substances or another medical condition. In general, the main form of therapy for PD is psychotherapy, with adjuvant pharmacotherapy. Due to a predisposition to instability and decompensation, individuals with PD are more likely to be admitted to a psychiatric hospital. With the passing of time, the frequency of PD diagnosis has been rising.

**Objectives:**

The study aimed to assess changes in the prevalence of PD diagnosis between the years 2009 and 2021 in the Psychiatric Central Clinical Hospital of the Medical University of Lodz (Poland) and the characteristics of admitted patients.

**Methods:**

This retrospective included 27097 records of patients admitted for psychiatric hospitalization between the years 2009 and 2021. The diagnosis of PD (F60 and F61) was based on ICD-10 diagnostic criteria. For analysis, both main, as well as coexisting diagnoses of PD were included. For the analysis patients were divided into subgroups based on age and legal gender.

**Results:**

We observed a statistically significant increase in the number of hospitalization of individuals with PD (6,94% in 2009 and 14,29% in 2021; p<0.0001). No rise in the frequency of F60 diagnosis was observed (4,56% in 2009 and 4,48%; p=0.973, while the diagnosis of mixed PD (F61) has greatly risen (2,38% in 2009 and in 9,81% in 2021; p=0.003), this growth was especially visible in men (1,62% in 2009 and 10,44% in 2021; p=0.007). In individuals above the age of 35 at the time of hospitalization significant growth in PD diagnosis was present (5,22% in 2009 and in 8,25% in 2021; p=0.003), similarly, PD increased in patients older than 65 (0,50% in 2009 and in 4,00% in 2021; p=0.003).

**Conclusions:**

In the past 13 years, there has been a great increase in the number of hospitalized individuals with PD, particularly the rise reflects growth in mixed PD diagnosis. Interestingly, in men, PD diagnosis is 4 times more frequent in 2021 than in 2009. The increase in the number of PD diagnoses in changing environment might be due to greater clinical vigilance of psychiatrists and a more in-depth diagnostic process, yet further analysis including data from the outpatient clinic is needed.

**Disclosure of Interest:**

None Declared

